# Dynamic kinematics of the glenohumeral joint in shoulders with rotator cuff tears

**DOI:** 10.1186/s13018-017-0709-6

**Published:** 2018-01-11

**Authors:** Naoya Kozono, Takamitsu Okada, Naohide Takeuchi, Satoshi Hamai, Hidehiko Higaki, Takeshi Shimoto, Satoru Ikebe, Hirotaka Gondo, Yoshitaka Nakanishi, Takahiro Senju, Yasuharu Nakashima

**Affiliations:** 1 Department of Orthopaedic Surgery, Graduate School of Medical Sciences, Kyushu University, 3-1-1 Maidashi, Higashi-ku, Fukuoka City, Fukuoka 812-8582 Japan; 2 Department of Life Science, Faculty of Life Science, Kyushu Sangyo University, 2-3-1, Matsukadai, Higashi-ku, Fukuoka City, Fukuoka 813-8503 Japan; 3 Department of Information and System Engineering, Faculty of Information Engineering, Fukuoka Institute of Technology, 3-30-1 Wajiro-higashi, Higashi-ku, Fukuoka City, Fukuoka 811-0295 Japan; 4 Department of Creative Engineering, National Institute of Technology, Kitakyushu College, 5-20-1 Shii, Kokuraminami-ku, Kitakyushu City, Fukuoka 802-0985 Japan

**Keywords:** Rotator cuff, Glenohumeral, Kinematics, Humeral translation, 3D-to-2D model-to-image registration techniques

## Abstract

**Background:**

No clear trend has emerged from the literature regarding three-dimensional (3D) translations of the humerus relative to the scapula in shoulders with rotator cuff tears (RCTs). The purpose of this study was to evaluate the kinematics of RCT shoulders using 3D-to-two-dimensional (2D) model-to-image registration techniques.

**Methods:**

Dynamic glenohumeral kinematics during scapular plane abduction and axial rotation were analyzed in 11 RCT patients and 10 healthy control subjects. We measured the 3D kinematic parameters of glenohumeral joints using X-ray images and CT-derived digitally reconstructed radiographs.

**Results:**

For scapular plane abduction, the humeral head center was positioned significantly more medially in shoulders with RCTs than in controls at 135° of humeral abduction (*p* = 0.02; RCTs versus controls: − 0.9 ± 1.6 versus 0.3 ± 1.3 mm). There was no significant difference in the superior/inferior translation of the humeral head center (*p* = 0.99). For axial rotation in adducted position, the humeral head center was positioned significantly more anteriorly in shoulders with RCTs than in controls at − 30° of glenohumeral external rotation (*p* < 0.0001; RCTs versus controls: 3.0 ± 1.7 versus 0.3 ± 1.5 mm).

**Conclusions:**

This study revealed the kinematics of shoulders with large to massive full-thickness RCTs: the humeral head center showed a medial shift at the late phase of scapular plane full abduction, and an anterior shift at the internal rotation position during full axial rotation. The kinematic data in this study, which describe the patterns of movement of shoulders with large to massive full-thickness RCTs, provide valuable information for future studies investigating glenohumeral translations in other pathological conditions of the shoulder. For clinical relevance, quantitative assessment of the dynamic kinematics of shoulders with RCTs might be a therapeutic indicator for achieving functional restoration.

## Background

Rotator cuff tears (RCTs) are a common shoulder disorder among elderly people and can cause shoulder pain, weakness, and decreased range of motion [1]. Patients with symptomatic RCTs often choose to undergo rotator cuff repair to relieve pain, improve function, and return to high-level activities [2, 3]. As patients’ function and ability are directly affected by joint kinematics, there is an interest in quantifying the shoulder kinematics of patients with RCTs. Of particular importance are preoperative shoulder mechanics during scapular plane abduction and axial rotation, as patients expect to perform these activities after rotator cuff surgery. Motion capture systems with reflective markers have been widely used for in vivo three-dimensional (3D) shoulder kinematics with RCTs. However, external markers attached to the skin might be affected by soft tissue artifact, producing substantial errors [4–6].

The accurate evaluation of kinematics under weight-bearing conditions has been achieved using 3D-to-two-dimensional (2D) model-to-image registration techniques [7–13]. With regard to the shoulder joint, several studies have recently reported shoulder kinematics using 3D-to-2D model-to-image registration techniques [14–20]. Our previous study demonstrated the in vivo kinematics of healthy shoulder joints with coordinated humeral and scapular dynamic movements [17].

Millett et al. [19] used 3D-to-2D model-to-image registration techniques and reported that subjects with RCTs demonstrated a dynamic inferior translation of the humeral head during scapular plane abduction. However, these findings are contrary to other published studies that found superior migration [21–24]. No clear trend has emerged from the literature regarding 3D translations of the humerus relative to the scapula in RCT shoulders during scapular plane abduction. Moreover, to the best of our knowledge, there have been no previously published reports on 3D translations of the humerus relative to the scapula in RCT shoulders during axial rotation, as measured by 3D-to-2D model-to-image registration techniques.

Several studies have reported on the movement of the external rotation of the humerus relative to the scapula during arm abduction [16–18, 25]. However, few reports have evaluated the external rotation of the humerus relative to the scapula in RCT shoulders during active abduction using 3D-to-2D model-to-image registration techniques [16].

The purpose of this study was to evaluate the kinematics of RCT shoulders during dynamic scapular plane full abduction and full axial rotation using 3D-to-2D model-to-image registration techniques. The following specific questions were addressed: (1) what is the 3D translation of the humerus relative to the scapula during dynamic scapular plane full abduction and full axial rotation; and (2) how large is the external rotation of the humerus relative to the scapula during dynamic scapular plane full abduction?

## Methods

**Fig. 1:**
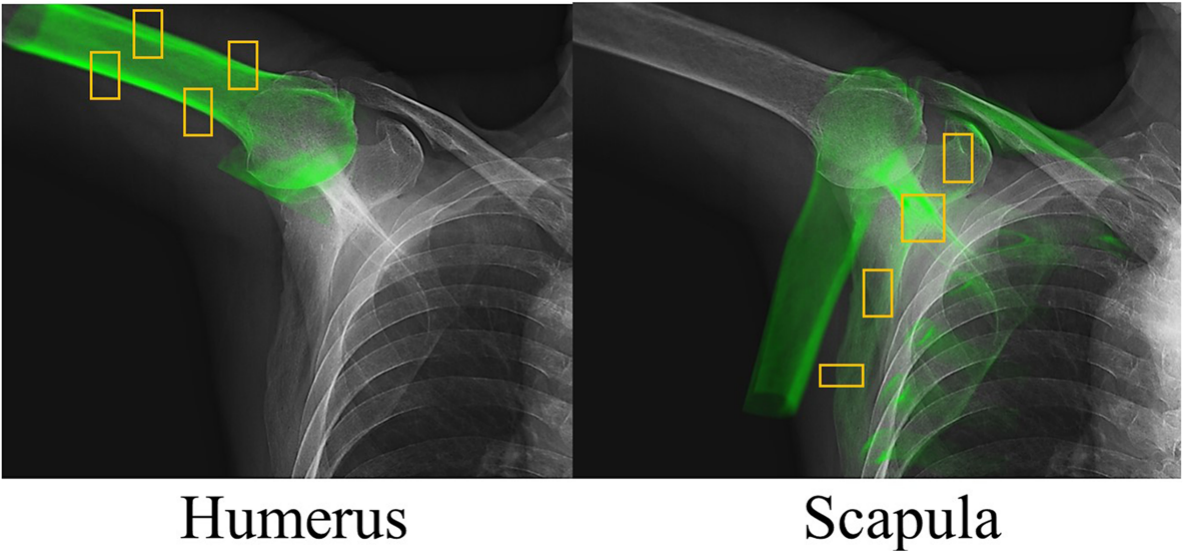
Shoulder computed tomography slices were used to create density-based digitally reconstructed radiographs of the humerus and scapula, which were projected onto the shoulder X-ray images

**Fig. 2:**
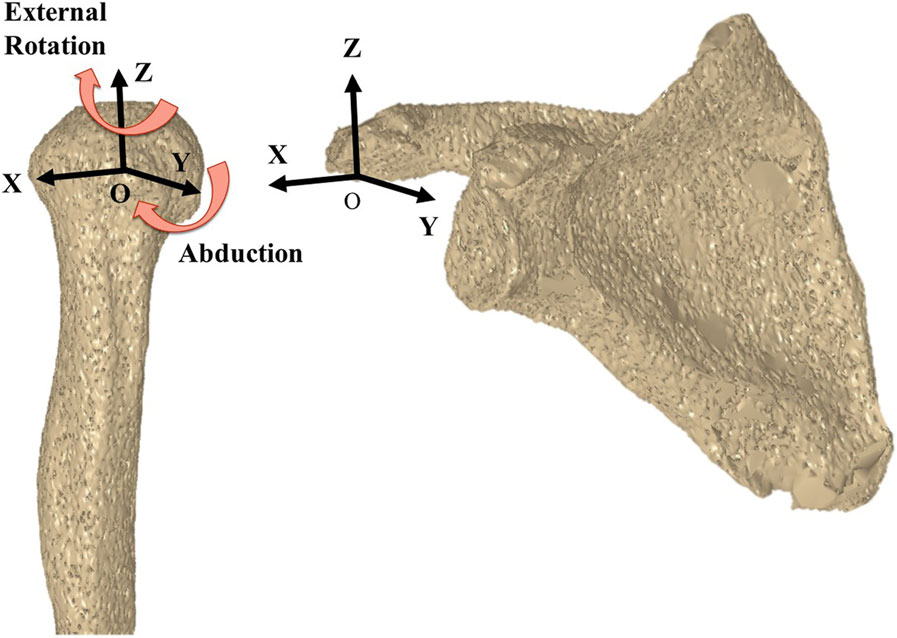
The anatomical coordinate systems of the humerus and scapula. Abduction of the humerus is defined as rotation about the *y*-axis. External rotation of the humerus is defined as rotation about the *z*-axis. Humeral translations were expressed relative to the scapular coordinate system. Superior (**+**)/inferior (−) = *z*-axis, anterior (**+**)/posterior (−) = *y*-axis, and lateral (**+**)/medial (−) = *x*-axis

This study included 21 subjects. All subjects gave their informed consent to participate in this Institutional Review Board approved study, and were informed of the risk of the radiation exposure required. Eleven of them were patients with a diagnosis of large or massive RCT who were scheduled to undergo rotator cuff surgery. The large to massive full-thickness RCTs were confirmed by magnetic resonance imaging (MRI). Moreover, the radiologic evaluation was performed using Hamada classification [26]. We excluded RCT shoulders with Grade 3 (subacromial arthritis), Grade 4A (glenohumeral arthritis, without subacromial arthritis), Grade 4B (glenohumeral arthritis, with subacromial arthritis), or Grade 5 (humeral head collapse, which is characteristic of cuff tear arthropathy). The size of the RCT was also measured intraoperatively according to the classification of DeOrio and Cofield [27], and the tears were categorized as either large (3–5 cm) or massive (> 5 cm). There were no superior labrum anterior and posterior lesions or long head of biceps tendon pathologies on preoperative MRI or later on intraoperative findings. Exclusion criteria were (1) neuromuscular disorders, (2) previous surgery, and (3) dysfunction of maximum active abduction of less than 120° in the analyzed shoulder. The 11 RCT patients consisted of 6 males and 5 females, with a mean age of 72 ± 5 years (range, 65–75); height, 158 ± 7 cm (range, 149–167); and weight, 57 ± 8 kg (range, 47–75). In addition, 10 healthy control subjects (all males; age, 32 ± 2 years [range, 30–37]; height, 174 ± 6 cm [range, 167–186]; weight, 70 ± 7 kg [range, 61–80]) with no history of shoulder injury, surgery, or symptoms were recruited for comparison, the results of which have been previously published [17]. The subjects were in a standing position in front of the flat-panel X-ray detector (FPD; Ultimax-I, Toshiba, Tochigi, Japan: 10 frames per second, image area size 420 mm [H] × 420 mm [V], and 0.274 mm × 0.274 mm/pixel resolution), and were positioned so that the coronal plane was perpendicular to the X-ray beam. Two shoulder motions of 4 s duration were recorded using a FPD. For dynamic scapular plane full abduction, abduction in the scapular plane was performed from the arm at the side to maximum abduction with the elbow extended fully and the arm rotated externally in the thumb-up position. For dynamic full axial rotation, axial rotation was performed in the adducted position with the elbow flexed at 90° from internal to maximum external rotation. Shoulder computed tomography (CT; Aquilion, Toshiba, Tochigi, Japan) was performed with a 512 × 512 image matrix, 0.35 × 0.35 pixel dimension, and 1-mm slice thickness spanning the entire humerus and scapula. A 3D gray-scale model was constructed in a virtual 3D space using the CT data, and a computer simulation of the radiographic process was carried out to generate a virtual digitally reconstructed radiograph (DRR) for each subject [8–10, 17]. The density-based DRRs were then compared with the serial X-ray images acquired using the FPD (Fig. 1). Correlations of the pixel values between the DRRs and real X-ray images were used to fine-tune the 3D model. Specifically, multiple image windows that spanned the bony structure were used for density-based image-matching analysis (Fig. 1) [8–10, 17]. Anatomical coordinate systems of the humerus and scapula were determined by a method that followed the International Society of Biomechanics standard [28], and were embedded in each density-based volumetric bone model according to the previous study (Fig. 2) [17]. The positions and orientations of the humerus were defined as humeral movements (flexion/extension about the *x*-axis, abduction/adduction about the *y*-axis, internal/external rotation about the *z*-axis) (Fig. 2) [17]. The *x*-, *y*-, and *z*-axes represented the lateral/medial, anterior/posterior, and superior/inferior axes (Fig. 2). Humeral translations were defined as motion of the origin of the humerus relative to the origin of the scapular coordinate system in the superior/inferior (S/I), anterior/posterior (A/P), and lateral/medial (L/M) directions (Fig. 2) [17]. For scapular plane full abduction, humeral translations (S/I, A/P, L/M) were normalized relative to the starting position of shoulder motion. For full axial rotation, humeral translations (S/I, A/P, L/M) were normalized relative to the neutral rotation of the glenohumeral joint in the adducted position with the elbow flexed at 90°.

An experiment to evaluate accuracy was performed on the humerus and scapula of a pig carcass [9, 10]. The humerus and scapula were fixed to a stage and rotated and translated to known values (0–10° rotation and 0–10 mm translation for the in-plane and out-of-plane directions, respectively) [9, 10]. For each position, three X-ray scans were acquired, and the 3D-to-2D model-to-image registration technique was performed for the X-ray images at each position to determine the orientations and positions of each bone. The measurement accuracy was evaluated using the root-mean-square (RMS) errors.

### Statistical analysis

Statistical analyses were performed using JMP software version 11.0.0 (SAS Institute Inc., Cary, NC). Values of *p* < 0.05 were considered to be statistically significant. For scapular plane full abduction, a 2-way repeated-measures analysis of variance (ANOVA) with independent factors of group (RCTs versus controls) and abduction angle was performed to detect differences in the humeral translations and glenohumeral external rotation. For full axial rotation, a two-way repeated-measures ANOVA with independent factors of group (RCTs versus controls) and external rotation angle was performed to detect differences in the humeral translations. When significant differences were found on ANOVA, post-hoc unpaired *t* tests were used for further significance testing. A power analysis indicated that a sample size of nine subjects per group would provide 80% statistical power for detecting a 0.7-mm difference in humeral translation between the groups. This assumes a probability value of *p* < 0.05 and a standard deviation of 0.3 mm.

## Results

### Scapular plane full abduction

**Fig. 3:**
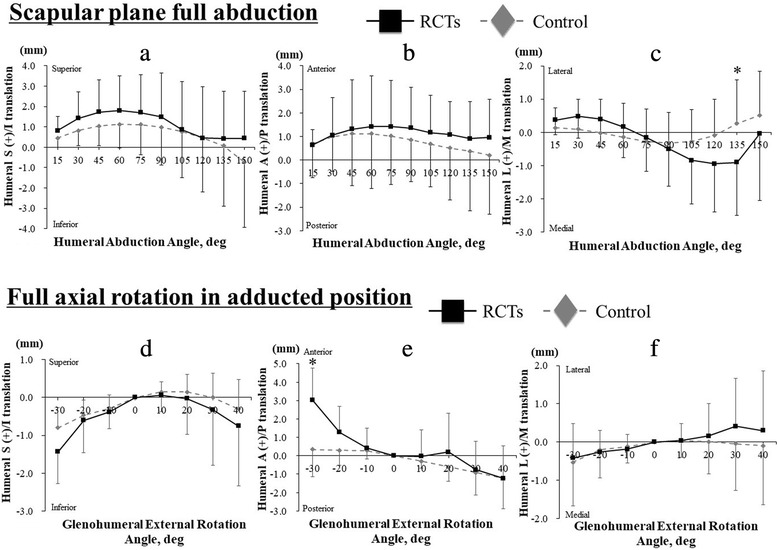
For scapular plane full abduction, **a** superior/inferior (S/I), **b** anterior/posterior (A/P), and **c** lateral/medial (L/M) translations of the humeral head are shown. For full axial rotation, **d** superior/inferior (S/I), **e** anterior/posterior (A/P), and **f** lateral/medial (L/M) translations of the humeral head are shown. Asterisk indicates statistically significant differences (*p* < 0.05)

**Fig. 4:**
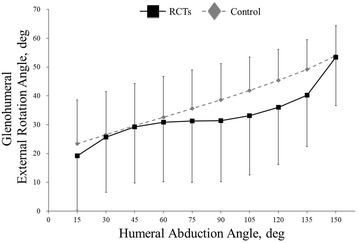
Glenohumeral external rotation during scapular plane abduction. There was no significant difference between the RCT and control groups

The S/I translation of the humeral head center did not significantly differ between the RCT and control groups (*p* = 0.99), even though the humeral head center was positioned slightly superiorly in shoulders with RCTs compared to controls at 15°, 30°, 45°, 60°, 75°, 90°, 135°, and 150° of humeral abduction (Fig. 3a). The A/P translation of the humeral head center did not significantly differ between the RCT and control groups (*p* = 0.98), even though the humeral head center was positioned slightly anteriorly in shoulders with RCTs compared to controls at 45° to 150° of humeral abduction (Fig. 3b). The humeral head center was positioned significantly more medially in shoulders with RCTs than in controls at 135° of humeral abduction (*p* = 0.02; RCTs versus controls: − 0.9 ± 1.6 versus 0.3 ± 1.3 mm) (Fig. 3c). Glenohumeral external rotation also did not significantly differ between the RCT and control groups (*p* = 0.17) (Fig. 4).

### Full axial rotation in adducted position

The S/I translation of the humeral head center did not significantly differ between the RCT and control groups (*p* = 0.63), even though the humeral head center was positioned slightly inferiorly in shoulders with RCTs compared to controls at − 30°, 20°, 30°, and 40° of glenohumeral external rotation (Fig. 3d). The humeral head center was positioned significantly more anteriorly in shoulders with RCTs than in controls at − 30° of glenohumeral external rotation (*p* < 0.0001; RCTs versus controls: 3.0 ± 1.7 versus 0.3 ± 1.5 mm) (Fig. 3e). The L/M translation of the humeral head center did not significantly differ between the RCT and control groups (*p* = 0.97) (Fig. 3f).

### Accuracy evaluation

The RMS errors for bone movement were as follows: 0.09 mm for in-plane translation, 0.05 mm for out-of-plane translation, and 0.17° for rotation of the humerus; and 0.07 mm for in-plane translation, 0.07 mm for out-of-plane translation, and 0.05° for rotation of the scapula.

## Discussion

This study had three major findings. First, the humeral head center was positioned significantly more medially in shoulders with RCTs than in controls at the late phase of dynamic scapular plane abduction. Second, the humeral head center was positioned significantly more anteriorly in shoulders with RCTs than in controls at internal rotation position during dynamic axial rotation in adducted position. However, we did not include RCT shoulders with a maximum active abduction of less than 120° or osteoarthritis in this study. Third, the analytical method allowed for the assessment of glenohumeral kinematics, achieving highly accurate measurements of humeral and scapular movements.

With regard to scapular plane full abduction, the humeral head center has been reported to translate superiorly in shoulders with RCTs during scapular plane abduction [21–24]. However, these previous studies employed a static measurement technique, and their results have not been validated by an in vivo dynamic 3D shoulder kinematic study. Conversely, Millett et al. [19] reported that the humeral head center translated inferiorly in shoulders with RCTs during scapular plane abduction, as measured by 3D-to-2D model-to-image registration techniques. In this study, the humeral head center was positioned slightly superiorly in shoulders with RCTs compared to controls at 15°, 30°, 45°, 60°, 75°, 90°, 135°, and 150° of humeral abduction; however, the difference was not significant in terms of S/I translation. The results reported by Millett et al. [19] are not consistent with those of this study. This discrepancy in humeral translation may be due to differences in RCT size. Millett et al. [19] analyzed patients with repairable full-thickness tears of the supraspinatus with or without 1-cm extension into the infraspinatus. Our results demonstrated that the humeral head center was positioned significantly more medially in shoulders with RCTs than in controls at the late phase. This kinematic change would be associated with the ratio of the contribution of the deltoid and the rotator cuff muscles [29–32]. The large to massive full-thickness RCTs would consistently require increasing deltoid muscle force for rotator cuff dysfunction during scapular plane abduction, which might be directed superiorly at the early phase and medially at the late phase. Additionally, we observed no significant difference in the external rotation of the humerus relative to the scapula during scapular plane full abduction between the RCT and control groups. Kijima et al. [16] reported that compared to healthy shoulders, shoulders with RCTs showed less external rotation of the humerus relative to the scapula in the early phase, as measured by 3D-to-2D model-to-image registration techniques. Their results are not consistent with the results of this study. This discrepancy in the observed glenohumeral external rotation may be because of differences in anatomical coordinate systems or RCT size [16, 18]. Our previous study [17] demonstrated that the humerus rotated 33.6° externally relative to the scapula in healthy shoulders during dynamic scapular plane full abduction, as measured by 3D–to-2D model-to-image registration techniques. The large to massive full-thickness RCTs seemed not to affect glenohumeral external rotation during dynamic scapular plane full abduction.

With regard to full axial rotation in the adducted position, our results showed that the humeral head center was positioned significantly more anteriorly in shoulders with RCTs than in controls at internal rotation position. Many studies reported that the rotator cuff muscles function as primary dynamic stabilizers to maintain a concentric reduction during rotation of the humeral head on the glenoid [33–38]. The subscapularis and infraspinatus/teres minor complex balance each other in the transverse plane. The large to massive full-thickness RCTs involve infraspinatus muscle impairment; therefore, the humeral head center was positioned anteriorly at internal rotation position.

With regard to the accuracy evaluation, this study demonstrated that the analytical method using density-based DRRs and a flat-panel X-ray detector is a non-invasive approach for the assessment of glenohumeral kinematics, achieving highly accurate measurements of humeral and scapular movements. Millett et al. reported that the accuracy measurements of the biplane fluoroscopy system averaged 0.3 mm for translational variables and 0.6° for rotational variables [19]. Also, Kijima et al. reported that the accuracy measurements of the biplane fluoroscopy system averaged 0.9 mm for translational variables and 1.3° for rotational variables [16]. The RMS errors in this study were equivalent to the results of these previous studies using biplane fluoroscopy [16, 19].

This study involves some limitations. First, we were limited by the small number of study subjects. A large number of shoulders may have revealed additional kinematic differences. However, the number of study subjects is similar to previous studies of shoulder kinematics using 3D-to-2D model-to-image registration techniques [15, 16, 18, 19]. Second, this study included only young male shoulders as healthy subjects owing to the demands of the study protocol with respect to X-ray surveillance and CT scanning. Third, the control group differed from the RCT group in terms of age and sex. The findings of this study should be interpreted with the understanding that these limitations may significantly bias the results. Fourth, we excluded RCT shoulders with a maximum active abduction of less than 120° or osteoarthritis. Therefore, our RCT cohort is not representative of the typical patients with RCTs encountered in daily clinical practice. Finally, we described the shoulder kinematics without holding hand weights in this study. Therefore, further studies are needed to investigate shoulder kinematic patterns while holding hand weights.

The clinical relevance of this study is that the quantitative assessment of the dynamic kinematics of shoulders with RCTs might be a therapeutic indicator for achieving shoulder functional restoration. We believe the kinematic data in this study would be useful in preoperative decision making and postoperative rehabilitation. The new findings in this study provide important information for the improved understanding of RCT shoulders.

## Conclusions

This study revealed the kinematics of shoulder with large to massive full-thickness RCTs: during scapular plane full abduction, the humeral head center showed a medial shift at the late phase, and during full axial rotation, the humeral head center showed an anterior shift at internal rotation position. There were differences in the 3D translations of the humerus relative to the scapula between RCT and healthy shoulders. The kinematic data in this study, which describes the patterns of movement of large to massive full-thickness RCT shoulders, provide valuable information for future studies investigating glenohumeral translations in other patient populations, such as those with partial or small to medium RCTs, impingement, instability, and arthritis.

## Abbreviations

2D: Two-dimensional
3D: Three-dimensional
A/P: Anterior/posterior
ANOVA: Analysis of variance
CT: Computed tomography
DRR: Digitally reconstructed radiograph
FPD: Flat-panel X-ray detector
L/M: Lateral/medial
MRI: Magnetic resonance imaging
RCTs: Rotator cuff tears
RMS: Root-mean-square
S/I: Superior/inferior
